# Feasibility, Acceptability, and Adherence with Short-Term HIV Preexposure Prophylaxis in Female Sexual Partners of Migrant Miners in Mozambique

**DOI:** 10.1097/QAI.0000000000001518

**Published:** 2017-08-01

**Authors:** Maria Lahuerta, Allison Zerbe, Rachel Baggaley, Joana Falcao, Laurence Ahoua, Pietro DiMattei, Fernando Morales, Isaias Ramiro, Wafaa M. El-Sadr

**Affiliations:** * ICAP at Columbia University, Mailman School of Public Health, New York, NY;; † Department of Epidemiology, Mailman School of Public Health, Columbia University, New York, NY;; ‡ HIV Department, World Health Organization, Geneva, Switzerland; and; § Ministry of Health, Maputo, Mozambique.

**Keywords:** preexposure prophylaxis, female partners of miners, migrant, Mozambique, short-term

## Abstract

Preexposure prophylaxis (PrEP) offers protection from HIV acquisition if taken as prescribed. We evaluated the feasibility, acceptability, and adherence with short-term PrEP among female sexual partners of migrant miners in Mozambique.

**Methods:**

HIV-negative female sexual partners of migrant miners were offered daily tenofovir/emtricitabine (TDF/FTC) for 6 weeks concurrent with miners' return home. Study visits occurred at baseline, week 4, 6, and 8. Dried blood spots (DBSs) were collected at week 4 and 6.

**Results:**

Seventy-four women (median age: 42 years) were enrolled, 95% reported having 1 sexual partner and 80% reported never or rarely using condoms. At baseline, 41% had never tested for HIV; 65% were unaware of partners' HIV status. Of all women, 72 (97%) initiated PrEP, 7 (9%) discontinued PrEP before week 6; only 1 due to adverse events. Missed doses in the last week were self-reported by 8% and 3% of women at week 4 and 6, respectively. Of 66 (89%) women with DBS at week 4, 79% had detectable tenofovir diphosphate (TFV-DP) and 44% had levels consistent with ≥4 pills/wk (≥700 fmol/punch). Of 63 (88%) women with DBS at week 6, 76% had detectable TFV-DP and 42% had levels consistent with ≥4 pills/wk.

**Conclusions:**

In this first study assessing the use of short-term PrEP, a high percent of female partners of migrant workers initiated PrEP and had detectable DP levels during follow-up. Further efforts are needed to enhance adherence to ensure protection from HIV acquisition. Short-term PrEP offers promise for populations who are at high risk of HIV during specific periods of time.

## INTRODUCTION

Oral preexposure prophylaxis (PrEP), if taken as prescribed, is effective in prevention of HIV acquisition.^1,2^ Consequently, in 2015, the World Health Organizatin (WHO) recommended the use of PrEP for prevention of HIV acquisition by people at substantial risk of HIV.^3^

Several studies have demonstrated that Mozambican miners working in South Africa and separated from their partners, often engage in high risk sexual activity—placing both themselves as well as their partners at home at risk of HIV acquisition.^4–6^ Gaza Province in southern Mozambique has the highest HIV prevalence in the country at 25%.^7,8^ This province also serves as a source of migrant labor for mines and industries in South Africa with male migrants returning for a long break in December (around Christmas) and a shorter break in March/April (around Easter). Data suggest that this migration contributes substantially to the epidemic in southern Mozambique.^9,10^ In a recent survey conducted in Gaza Province, 35% of migrant miners stated that they had engaged in sex outside of their marriage in the last 12 months and overall 46% reported never using condoms.^11^

Given the specific circumstances of this population and high risk of HIV acquisition, novel approaches to HIV prevention are urgently needed. Mathematical models suggest that provision of time-limited PrEP to partners of miners could substantially reduce the cost per infection averted.^12^ However, to date, no study has assessed the feasibility, acceptability, and adherence with short-term PrEP during periods of heightened risk of HIV exposure. We evaluated the feasibility, acceptability, and adherence with short-term daily oral PrEP among HIV-negative female partners of male miners in Gaza Province, Mozambique.

## METHODS

### Study Setting and Population

The study took place between March and April 2016 in Chibuto and Xai–Xai, 2 districts of Gaza Province, in Mozambique. Participant recruitment and follow-up were conducted at TEBA offices, a labor agency that employs men in Mozambique and deploys them to work in mines in South Africa. Women were included in the study if they were 18 years of age or older, a female partner of a miner employed by TEBA, had a negative HIV test result per national HIV testing algorithm, had a serum creatinine below the upper limit of normal and calculated creatinine clearance >60 mL/min and provided written informed consent. Women were excluded if they had an hepatic alanine aminotransferase more than 2 times the upper limit normal; had symptoms of acute HIV infection; were pregnant or breastfeeding; had a history of kidney or other chronic disease; or if the miner partner was not coming to visit during Easter break in March/April 2016.

### Study Procedures

Female partners of miners were informed of the study and, if interested, were referred to the study team. At baseline, eligible women who provided written informed consent were interviewed for sociodemographics, risk behaviors, HIV knowledge and attitudes, HIV prevention knowledge, and attitudes including the use of PrEP. Participants were counseled regarding how to take PrEP, the importance of adherence for PrEP to be effective, HIV risk reduction, condom use, and family planning.

Participants were instructed to take 1 tablet of tenofovir/emtricitabine (TDF/FTC 200 mg/300 mg) orally daily for the 2 weeks before partner's return, throughout the 2 weeks that their partner was home, and for 2 weeks after the partner returned to mines (total of 6 weeks). Participants were given a 5-week supply of TDF/FTC at baseline and a 3-week supply at week 4. Women were asked to return to the study office if they experienced any side effects, social harms, or symptoms of acute HIV infection.

At week 4 and 6, participants completed a follow-up questionnaire on behaviors, adherence, social harms, and adverse events. Additional counseling regarding PrEP adherence, HIV risk reduction, condom use, and family planning was provided at follow-up visits. Participants were tested for HIV and alanine aminotransferase. Dried blood spots (DBS) were collected. Two weeks after PrEP cessation (week 8), participants were assessed for adverse events and any social harm.

### Adherence Measurements

Adherence with PrEP was ascertained using 3 methods: self-report, pill count, and tenofovir diphosphate levels in DBS. Pill count adherence was determined by counting the number of pills returned at week 4 and 6 visits and comparing with the expected number to have been used. DBS samples were assessed in a 3-mm DBS punch for tenofovir diphosphate levels using a validated liquid chromatography/tandem mass spectrometry method.^13–15^ The dynamic range of the assay was 25–6000 fmol per sample for TFV-DP.

### Ethical Considerations

The protocol was approved by the Mozambique National Bioethics committee and the Institutional Review Boards at the WHO and Columbia University Medical Center.

## RESULTS

### Study Participants

Between February and March 2016, 97 women were screened for study eligibility, with 23 excluded. Primary reasons for ineligibility were being HIV-positive (9) and miner partner not returning during the planned study period (2).

A total of 74 women were enrolled in the study, with a median age of 42 years [interquartile range:37–50], the majority had at least some primary education (87%), most worked in agriculture or at home (78%) and 92% were married or living together with a partner (Table 1). More than one-third (41%) had never previously tested for HIV, whereas 33% had received an HIV test within the past 6 months. Regarding the partner's HIV status, 8% knew their partner was HIV positive, 27% reported their partner was HIV negative, and 65% did not know. No participant was diagnosed with HIV during follow-up.

**TABLE 1.**
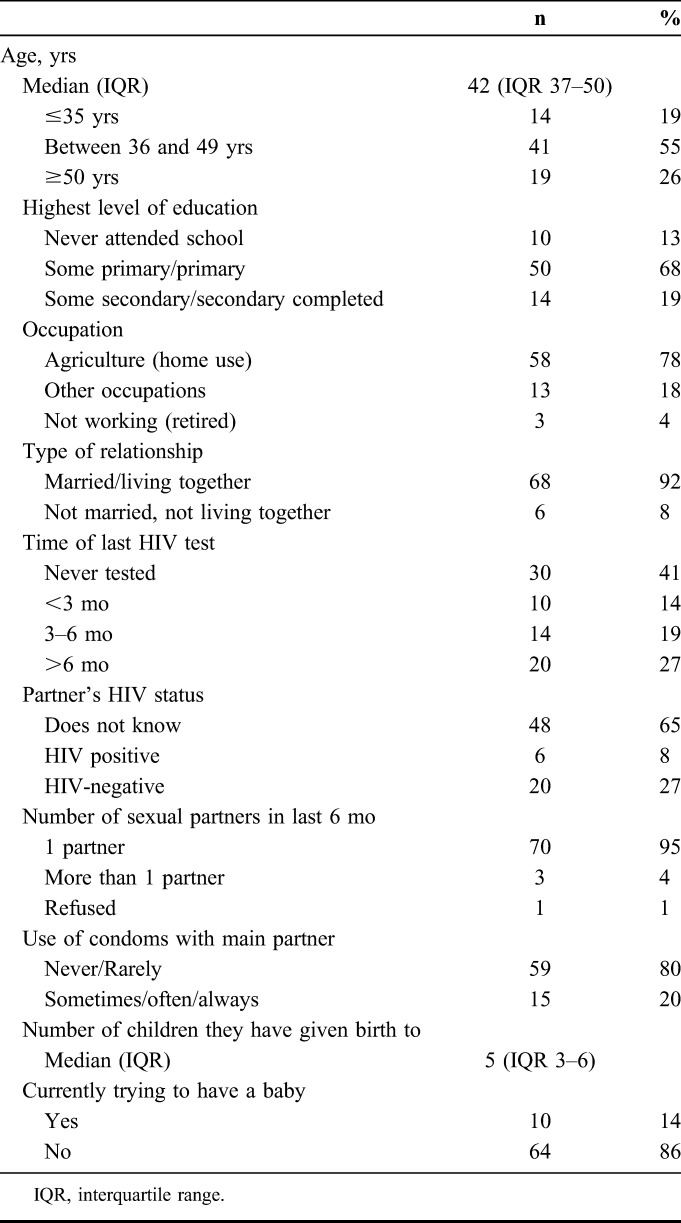
Characteristics of Study Participants at Baseline (N = 74)

### Sexual Behaviors

At baseline, the majority (95%) reported having 1 sexual partner and 80% reported never or rarely using condoms (Table 1). Women reported a median of 5 (interquartile range: 3–6) births; 14% were currently trying to have a baby and 20% were using some family planning method. At week 4, all reported having sex with their partner since last visit, with 89% never or rarely using condoms. There were no changes reported in condom use during follow-up. During follow-up, only 1 woman reported having other sexual partners besides the main miner partner.

### Uptake and Duration of PrEP Use

Of the 74 participants enrolled in the study, 72 (97%) initiated PrEP. One woman did not initiate because of fear and pressure from her family, whereas the other was taking traditional medicine. Overall, 66 (89%) reported taking PrEP for 4 weeks, 64 (86%) reported taking PrEP for 6 weeks and completed the week 6 visit, and 64 (86%) completed the week 8 visit (2-weeks after discontinuation). Seven participants (9%) discontinued PrEP before week 6 with only 1 due to side effects. Other reasons for discontinuing PrEP included: lack of time to return to the study office, traveling with the partner, and fear of family finding out. One woman was lost to follow-up since she was traveling outside of the country.

### Adverse Events

The most common adverse events reported at week 4 were headache, fatigue, or dizziness (44%) followed by digestive problems (32%). No severe adverse events were reported. The proportion reporting adverse events decreased at week 6 visit, with 7 (11%) reporting headache, fatigue or dizziness, and 3 (5%) reporting digestive problems. Only 1 woman indicated stopping PrEP due to adverse events.

### Adherence With PrEP

Self-reported adherence for the previous day was 98% and 97% at week 4 and 6, respectively, whereas 92% and 97% of the women reported having missed 1 or more doses in the previous 7 days, respectively (Table 2). At week 4 visit, 15 women (23%) reported always taking PrEP, as instructed, this percent increased to 69% by week 6. Based on the pill count data, the majority (92% and 90% at week 4 and 6, rspectively) used more than 90% of expected doses.

**TABLE 2.**
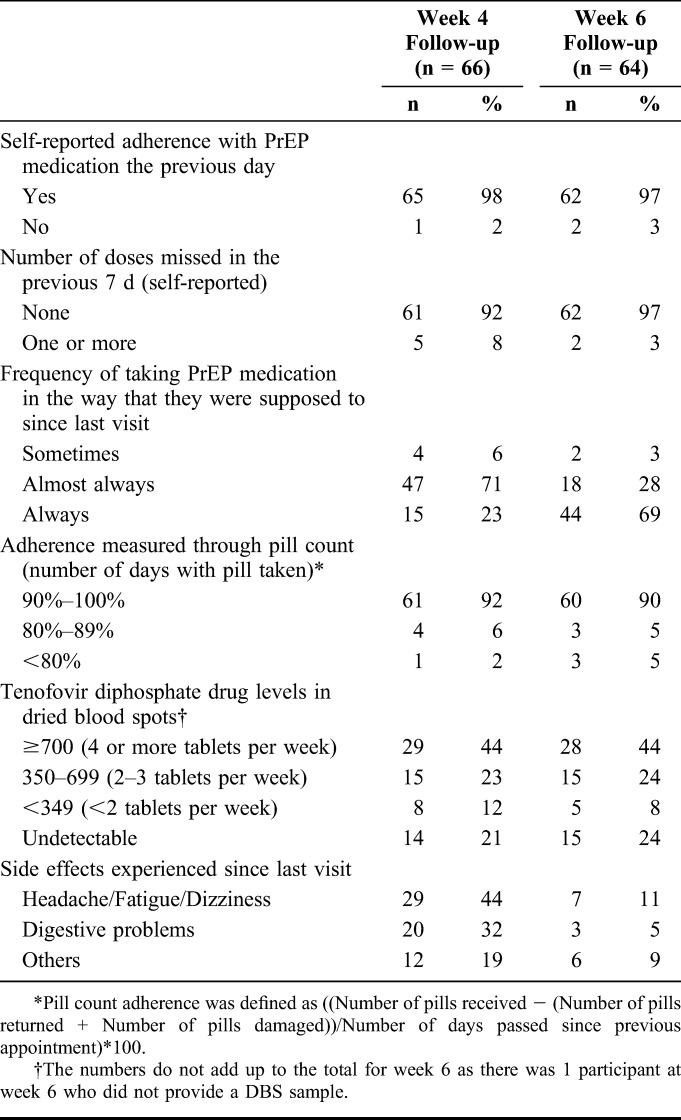
Self-Reported Adherence, Drug Levels in Plasma, and Side Effects at Week 4 and 6

Of the 66 (89%) women with DBS at week 4, 79% had detectable levels of TFV-DP. At week 4, 44% had levels consistent with ≥4 pills/wk (TFV-DP levels ≥700 fmol/punch), 15 (23%) had levels consistent with 2–3 tablets per week (350–699 fmol/punch), 8 (12%) had levels consistent with less than 2 tablets per week (<349 fmol/punch), and 14 (21%) had undetectable drug level. Of the 63 (88%) women with DBS at week 6, 76% had detectable levels of TFV-DP, with similar concentration distribution as at week 4 visit.

### Acceptability of PrEP

The main reason reported by the women for participation in the study was to protect themselves from getting HIV infection through their partner's sexual behavior. The majority disclosed study participation to a family member or friend (83% and 88% at week 4 and 6, respectively). However, 12% and 14% informed their partner about their study participation. No social harms were reported during the study. By the end of the study, 98% of the 64 women interviewed at week 8 were willing to continue taking pills everyday to protect themselves from HIV, and all indicated that they would recommend PrEP to a family member or friend.

## DISCUSSION

In this first study evaluating the use of short-term PrEP, we found that such use by female partners of migrant workers in Mozambique was feasible and acceptable in this high HIV prevalence setting. Uptake of PrEP was high, a large proportion of women had detectable TDF-DP levels during follow-up and the majority completed the PrEP course for the 6 weeks with almost half having levels consistent with ingestion of 4 or more pills in the previous week. Although reporting of adverse events was common early during PrEP use, this ameliorated during continued use and was largely not associated with discontinuation of PrEP. The high acceptability and uptake are consistent with findings from our previous qualitative study in this population.^16^

Our study focused on a population at substantial risk of HIV given that their main partners are migrant miners and based on findings from previous studies of high proportions of sex outside of their marriage and low condom use among these miners.^11^ Despite the promising results of PrEP as an HIV prevention strategy, few studies have evaluated the use of open-label PrEP among women in sub-Saharan Africa beyond the initial placebo-controlled clinical trials.^17–19^ Previous placebo-controlled studies that offered PrEP to women found adherence ranged from 20% to 80%, and a meta-analyses indicated that higher adherence of drug is needed to protect women from HIV infection.^19–22^ In this study, the proportion of participants with drug detected in the blood at week 6 (76%) were higher than that found in 2 placebo-controlled randomized PrEP trials, FEM-PREP (less than 40%) and VOICE studies (29%), among women and similar to those found in the open-label daily PrEP in the HPTN 067 (ADAPT) study (79%).^20–22^ The higher adherence observed in this study might be due to the short-term duration of follow-up. Despite the high self-reported adherence, 44% of participants had an evidence of ingestion of 4 or more pills in the previous week, thought to provide protective drug levels. Our results highlight the challenges of assessing and supporting adherence to daily PrEP and the need to enhance adherence counseling.^23^

In this study, very few women (14%) disclosed to their partners that they were taking PrEP, which could have impacted adherence given that partner awareness and support have been noted as key facilitators for adherence among women taking PrEP.^24–27^ Although women reported that it might not be advisable to take PrEP without a partner's approval, they also believed it was necessary to do so to protect themselves from HIV infection.^16^ This highlights the importance of supporting women in disclosing PrEP use, if they so desire. It is equally important to support women who choose not to disclose to their partners, providing them with the support they need to adhere with PrEP and avoid HIV infection. It is encouraging that the majority disclosed study participation to friends or family members. Finally, another important finding from our study is the substantial percent (9.3%) of women found to be HIV positive at screening who were all referred for HIV treatment. This highlights that the availability of PrEP may contribute to an increase in HIV testing coverage among vulnerable populations.

Given the small sample size and recruitment strategy, the findings may not be representative of all female partners of miners in Mozambique. However, this study involved a novel method for the use of oral PrEP in a population at substantial risk of HIV infection that has hitherto not been prioritized. Additional implementation science research is needed to build on our encouraging findings, determine how this intervention can be best implemented, and evaluate different types of adherence support to optimize the promise of PrEP in this population.
